# Chemsex, Identity and Sexual Health among Gay and Bisexual Men

**DOI:** 10.3390/ijerph191912124

**Published:** 2022-09-25

**Authors:** Rusi Jaspal

**Affiliations:** Vice-Chancellor’s Office, University of Brighton, Brighton BN2 4GJ, UK; rusi.jaspal@cantab.net

**Keywords:** chemsex, gay and bisexual men, identity, minority stress, mental health, sexual health

## Abstract

This article focuses on some of the social, cultural and psychological aspects of drug use in sexualized settings in gay and bisexual men (referred to as “chemsex”). Using a narrative review approach, the article examines previous empirical research in this area and presents a novel theoretical approach for understanding and predicting chemsex behavior. Tenets of identity process theory from social psychology are drawn upon to offer an integrative theoretical framework within which the social, cultural and psychological underpinnings of chemsex can be collectively examined. Existing empirical research suggests that gay and bisexual men may experience sexuality-related stressors that can undermine feelings of self-esteem, self-efficacy, continuity and positive distinctiveness. Identity process theory examines how individuals react to threats to identity brought about by these stressors. In response to identity threat, gay and bisexual men may engage in chemsex as a coping response that encompasses and facilitates various, largely maladaptive, coping strategies and tactics. The more chemsex is perceived as enhancing identity processes and as averting identity threat, the more central it is likely to be to the identities of participants. The centrality of chemsex to one’s identity may preclude self-withdrawal from the practice. Several directions for future research are presented based on existing work on chemsex viewed through the lens of identity process theory. These should form the basis of future empirical research in the sphere of sexual health among gay and bisexual men and the outcomes of this research should inform policy and practice in this area.

## 1. Introduction

There have been significant positive developments in relation to the rights of lesbian, gay, bisexual and trans (LGBT) people. Survey data show that attitudes toward LGBT people are improving [1]. The Equality Act 2010, which provides LGBT people with protection from discrimination, victimization and harassment on the basis of their sexual orientation or identity in the workplace, is a case in point. However, in many respects, LGBT people remain a marginalized population. Overt prejudice and discrimination that preceded the Equality Act 2010 have gradually morphed into more subtle, and often difficult to measure, “microaggressions” [2]. Stressors of this kind underpin the mental health and sexual health inequalities that persist in the LGBT population [3]. 

Mental health is defined as “a state of well-being in which the individual realizes his or her own abilities, can cope with the normal stresses of life, can work productively and fruitfully, and is able to make a contribution to his or her community” [4]. Sexual health refers to well-being across various interconnected domains (namely, physical, mental and emotional) in relation to one’s sexuality, rather than to the absence of sexually transmitted infections (STIs) alone [5]. There is evidence that LGBT people and gay and bisexual men, in particular, face sexual health inequalities when compared to their heterosexual counterparts [6]. These inequalities include a greater risk of exposure to human immunodeficiency virus (HIV) as well as to other STIs. It is clear that some behavioral factors, such as the relatively high prevalence of substance use in gay and bisexual men, are driving sexual health inequalities. Substance use in sexualized settings (or “chemsex”), in particular, is an important driver [7]. 

Accordingly, this article focuses on some of the social, cultural and psychological aspects of chemsex. The specific objectives are:

1. To examine both the potential motivations of gay and bisexual men who engage in this practice and its possible health consequences;
2. To identify protective factors that may limit the risk of engagement in problematic chemsex.

Complementing public health approaches to health behavior, tenets of identity process theory [8,9] are drawn upon to offer an integrative framework within which the social, cultural and psychological underpinnings of chemsex can be collectively examined. Based on previous empirical research derived from a narrative review approach, a series of directions for future research are presented. It is recommended that these should form the basis of future empirical research in the sphere of chemsex and sexual health among gay and bisexual men and that the outcomes of this research should inform policy and practice in this area. 

## 2. Method

In order to generate a corpus of outputs for review in this article, a systematic search of the electronic library databases PubMed and PsycINFO was conducted in July–August 2022. The aim was to identify original research papers that examined social representations of chemsex, the role of identity in chemsex, stressors that may prompt gay and bisexual men to engage in the practice and the relation between chemsex and sexual health. The search was not restricted to a particular time period, although there was an attempt to include the most recent data wherever possible. Search terms included: “chemsex” and “drug use in sexualized settings” in combination with “identity” or “stressors” or “sexual health”. These search terms were applied to titles in the databases and all searches were restricted to studies published in English, acknowledging that, as a global phenomenon, there is also published research in other languages. Grey literature was not included in this review. Studies were selected where at least one of the study populations was gay and/or bisexual men. All study designs were eligible. Final selection was based on relevance to identity, stressors and/or sexual health. The selection was discussed by the author with another researcher in this area until consensus was reached about the articles to be included in the review. A narrative review approach was utilized [10,11]. In this article, tenets of identity process theory [8,9] are drawn upon to review the available literature and to develop tentative hypotheses about social, cultural and psychological aspects of the practice. This is intended to inform the development of a systematic and theoretically informed research program in this key public health area.

## 3. Social, Cultural and Media Representations of Chemsex

Substance use in sexualized settings is by no means a novel practice. It has occurred for many centuries and in many different social groups [12]. However, the specific practice of chemsex is relatively novel, having been acknowledged and discussed in the UK since around 2011. The physiological effects of engaging in chemsex have been outlined elsewhere [13]. On the whole, the substances used in chemsex sessions (e.g., mephedrone, γ-hydroxybutyrate (GHB), γ-butyrolactone (GBL) and crystallized methamphetamine) tend to have a disinhibiting effect on users, enabling them to feel more sexually aroused, have longer and more intense sexual encounters with multiple partners and overcome common social, psychological and physiological barriers to sex (e.g., feeling self-conscious, worrying about one’s HIV status, experiencing pain during sex). Overall, the substances reportedly enable users to think, feel and do things that they would not while sober. Indeed, engagement in chemsex may transiently enhance a person’s sense of control and competence in their lives, that is, their self-efficacy.

As Stuart [14] has convincingly argued, chemsex has unique socio-cultural characteristics that render it a distinctive practice. More specifically, chemsex involves a specific set of substances; it tends to be organized on geospatial social networking applications for gay and bisexual men; and it has emerged against a socio-cultural backdrop of homonegativity, the desire for distinctiveness from the heterosexual majority and the quest for a sense of collectivity with other gay men, especially in view of a waning sense of community in this population [15]. Crucially, as argued in this article, chemsex engagement may constitute a response to one’s lived experience of stressors, reflecting an overarching coping response (consisting of specific strategies and tactics) to enhance one’s overall sense of identity. Stuart [14] (p. 9) warns against the inappropriate cultural appropriation of the term “chemsex” to describe the use of substances in sexualized settings in other populations given that the term “helps gay communities to name and identify a unique syndemic of behaviors and circumstances, so that community responses can be mobilized.”

Many scholars and commentators have focused on the potential harms associated with the practice of chemsex, especially for one’s sexual health. This specific focus has led some to challenge what they perceive as the growing stigmatization of chemsex, perhaps initiated in the academic (and especially public health) literature but certainly accelerated in the mainstream press. Hakim [15] has argued that a discourse of moral panic has emerged in relation to chemsex in the British press, with some media commentators constructing the practice as self-destructive for gay and bisexual men and as emphasizing its association with HIV infection, addiction and other severe risks to public health. Similarly, Heritage and Baker [16] found, in their corpus linguistics analysis of the mainstream British press, that extreme criminal cases involving chemsex were the main focus of reporting. These negative media representations operate as part of a broader system of factors that stigmatize aspects of gay men’s (sexual) lives, for example, [17,18,19]. 

Yet, it must be acknowledged that the epidemiological data do show a strong association between engagement in chemsex and risk practices, including a higher rate of HIV infection in those who practice chemsex [7,20]. Moreover, there have been many reports of sexual assault and other illegal activity in chemsex environments [21]. Notwithstanding the empirical associations between chemsex, sexual risk and adversity, dominant social representations of chemsex tend to obscure the reality that some gay and bisexual men who engage in chemsex manage their engagement in this practice effectively and do not go on to develop significant health problems [22]. Furthermore, some come to engage in chemsex not due to some prior trauma but rather for recreational reasons [23]. It is important not to lose sight of the diversity that characterizes gay and bisexual men who engage in chemsex. 

The way in which chemsex is perceived is closely associated with the way in which it is communicated. The social, cultural and psychological ramifications of science and health communication are considerable. The pathologizing of gay and bisexual men who engage in chemsex may lead to increased stigma surrounding the practice and, thus, decreased help-seeking among those who need support [24]. After all, people tend to “other” risk in an attempt to protect their self-concept and especially their self-esteem [25]. Their focus on distancing the notion of risk from their identities may constitute a means of maintaining continuity of self-construal.

Notwithstanding these observations, it is unclear how significant a problem chemsex actually is. Understanding the prevalence of chemsex is no easy task. Put simply, empirical studies appear to be telling us different things. Some indicate that chemsex is relatively uncommon, while others indicate its high and increasing prevalence [26]. These mixed findings can be explained. Studies rely on self-report data, which are never completely reliable—social stigma and social desirability biases may lead some to conceal their chemsex participation. Sampling biases in our own research may lead us to focus on specific subgroups of gay men who are more or less likely to be engaging in chemsex. For instance, sexual health research conducted largely in the context of sexual health clinics, which gay and bisexual men who are relatively informed about sexual health are more likely to attend compared to those who are not, often suffers from sampling bias. Moreover, there are notable differences in chemsex prevalence by geographical context—in the United Kingdom, major cities, such as London, Manchester and Liverpool, tend to have a higher prevalence of chemsex than smaller cities and rural areas in the country, as does England compared to the other nations of the United Kingdom [27].

## 4. Public Health Approaches to Health Behavior

Public health research has been guided by many different theoretical approaches. The ecological models of health behavior [28] tend to focus on the environmental and policy contexts of behavior and draw on social and psychological factors in doing so. They acknowledge multiple levels of influence, including intrapsychic, interpersonal, community/institutional factors and public/policy factors, thereby attempting to provide a more comprehensive framework for understanding health behavior, including risk-taking. Research focusing on the social determinants of health append particular importance to the role of social and socio-economic factors, such as one’s income, education and neighborhood, in shaping health behaviors and outcomes [29]. These approaches have been used to examine HIV risk and health outcomes in those living with HIV [30]. While acknowledging multiple levels, the models emphasize the social and institutional determinants, often providing limited attention to intrapsychic factors. 

Psychological research into health risk behaviors has, conversely, tended to focus on these intrapsychic factors, such as the significance of personality traits. Certain personality traits and profiles, such as sensation-seeking and impulsivity, have been found to be associated with a greater likelihood of engaging in risk behaviors, for example, [31]. Models of behavior change, such as the theory of planned behavior, emphasize the significance of social norms, behavioral intention and self-efficacy, in determining outcomes [32,33]. The concept of identity has been relatively less researched in relation to risk though, see [34]. Yet, it seems obvious that how one views oneself will guide one’s perceptions, emotions and actions in relation to risk. It is important to note that all these factors operating at multiple levels play a role in explaining and predicting health behaviors and outcomes. Psychological approaches to health behavior should work in conjunction with those in the public health sphere. An approach focusing on the significance of identity can enable us to reconcile these approaches [8].

## 5. The Significance of Identity

Identity process theory [8,35,36] focuses on the construction, management and defense of identity in the face of constant change. The theory was developed to examine the “blackbox” of identity by focusing not only on the content of identity (i.e., its constituent elements) but rather on how people react when identity undergoes some form of change. It posits that people construct their identity by engaging in two universal processes:

- *Assimilation-accommodation* refers to the absorption of novel stimuli in the identity structure and the subsequent changes that occur within that structure to accommodate its assimilation. For instance, a gay man who learns that he is HIV-positive may absorb knowledge of his new HIV status into his identity and this may also precipitate structural changes, such as the attenuation of his family identity as an avoidance strategy especially if his family is unaccepting [9]. This can be attributed to the shame that may accompany the diagnosis, especially on HIV status disclosure.
- *Evaluation* refers to the meaning and value that the individual appends to identity elements, that is, pre-existing identity content as well as the newly assimilated-accommodated elements. For instance, HIV does not mean the same thing to everyone—for some, it is negatively evaluated to the extent where it may not even be acknowledged while, for others, it may be construed as a positive life event that precipitated favorable changes in one’s life [37]. To that extent, the evaluation process is subjective and will, in part, depend on the social representations (that is, versions of social reality) to which the individual is exposed and which the individual actually accepts and internalizes [38].

According to the theory, the identity processes operate in ways that satisfy four main identity principles or motives:

- *Self-esteem* refers to one’s personal and social worth, that is, the extent to which one values oneself and feels valued by others. Self-esteem is an important principle of identity that affects many spheres of life, including perceptions of oneself and others, decision-making and behavior. However, research shows that self-esteem is not necessarily a “prime” principle for everybody as it operates in conjunction with the other identity principles to produce a positive sense of self [39]. There is evidence that gay and bisexual men experience challenges to their self-esteem due to stressors associated with their sexuality [40]. Moreover, stigma research, for example, [41], would lead us to hypothesize that engagement in stigmatized behaviors, such as chemsex, may also undermine a person’s self-esteem.
- *Distinctiveness* reflects the perception that one is sufficiently differentiated from other people and that this differentiation is positively evaluated. Two key points should be made in relation to distinctiveness. First, it is possible to feel negatively distinctive, which indeed arises on the basis of a stigmatized identity element (e.g., one’s sexuality, HIV status, chemsex participation). This would not satisfy the distinctiveness principle and would in fact probably also damage a person’s sense of self-esteem. Second, it has been noted that achieving a sense of acceptance and inclusion in valued social groups (or a sense of belonging) is also an important psychological need. Thus, an “excessive” sense of distinctiveness would not satisfy the need for belonging. This balancing of distinctiveness and belonging is at the heart of the optimal distinctiveness theory [42]. Hickson [43] notes that engagement in chemsex may constitute a rejection of the normalization of homosexuality in mainstream society and, thus, provide a sense of distinctiveness.
- *Continuity* refers to the psychological thread that an individual establishes between their past, present and future amid inevitable personal and social change. Despite this change, the individual must continue to believe that a factor or set of factors unify their past, present and future. This will often be a major identity element, such as a particular group membership, personality trait, or physical characteristic. Uncertainty about the future can easily undermine a person’s sense of continuity as they are no longer assured that their identity will remain temporally connected. For instance, while disclosure of one’s sexual identity is generally deemed to be psychologically beneficial, it can also represent a hazard to one’s sense of continuity [44]. After all, people do not always react positively to one’s coming out and, even if they do, the nature of one’s relationships may change—for better or for worse—potentially leading the individual to lose that crucial psychological thread between past, present and future.
- *Self-efficacy* is the perception of control and competence in one’s life. Crucially, this does not refer to any objective sense of efficacy in life but rather to the individual’s own appraisal of their efficacy. Self-efficacy is closely linked to self-esteem in that a person who feels in control of their life tends to derive greater self-worth and indeed a person with higher self-worth tends to have greater belief in their own efficacy [45,46]. In the context of chemsex, the perception that one can do things that one would not ordinarily feel able to do when sober may transiently enhance one’s sense of self-efficacy. Conversely, the inability to disengage from chemsex when one recognizes that this is hazardous to one’s social and psychological well-being may stimulate feelings of impotence in the same way that attempting, but failing, to quit smoking might [47].

Given that self-esteem, positive distinctiveness, continuity and self-efficacy are “desirable end-states for identity” [8] (p. 24), individuals actively strive to maintain high levels of these principles primarily through their engagement with the aforementioned two identity processes. For instance, it has been shown that people tend to see as more central to their identity those elements that satisfy one or more of these principles [48]. In other words, when asked to rate how important a particular identity element (e.g., being a chemsex participant) is to them, the individual will rate as more central those that play a greater role in satisfying the identity principles. Bardi, Jaspal, Polek and Schwartz [49] found that individuals differed in the extent to which they valued each of the identity principles and that there was a significant correlation between their own personal values (e.g., conservation) and the identity principles that they prioritized over others (e.g., continuity in the case of those valuing conservation). Thus, it could be said that individuals possess some “prime” identity principles that align with their personal and cultural value orientations.

A key proposition in identity process theory is that, when the processes of assimilation-accommodation and evaluation fail to comply with the principles of self-esteem, positive distinctiveness, continuity and self-efficacy, the individual will experience identity threat. When threatened, the stability of identity itself is undermined. The experience of identity threat, though commonplace, is harmful to psychological well-being. Of course, the extent of the harm will depend on the extent and severity of the threat itself. Three general hypotheses can be offered. First, when multiple principles of identity are obstructed, the threat is likely to be more severe [50]. Second, when the coping options (discussed later in this article) available to the individual are limited, the threat is likely to be more harmful [8]. Third, when the threat is long-standing and unresolved, its resurgence in any given context can feel especially acute [51]. Gay and bisexual men may be especially susceptible to identity threat due to exposure to stressors relating to their sexuality. 

## 6. Exposure to Stressors

Minority stress theory [3] has become an important theoretical framework for examining the impact of “stressors” associated with one’s minority status. Stressors refer to events, situations and societal perceptions that can lead to psychological stress. In minority stress theory, it is noted that exposure to these stressors can precipitate poor mental health.

The theory refers to distal and proximal stressors and notes their differential impact on the psychological health of LGBT people. Distal stressors are prejudice events that are external to the individual, such as the experience of “microaggressions” or exclusion due to their sexual orientation. Proximal stressors reflect the individual’s internal response to these events, such as internalized homonegativity, that is, the acceptance of negative social representations of their sexual orientation. It has been found that these distinct types of stressors may contribute differently to depressive and anxious symptomatology in sexual minorities [52,53]. In order to understand how and why particular types of stressors affect mental health, it is important to understand the individual’s sense of identity. A stressor that targets a more central identity element (that is, one that plays a greater role in satisfying the identity principles) may be more threatening for identity.

It is argued in minority stress theory that sexual minorities face stress that is unique to them and additive (over and above the habitual stressors that people from the general population face); chronic as it is rooted in long-standing and relatively stable social and cultural structures; and socially based because it arises from social processes, institutions and representations rather than individual characteristics. The theory identifies three processes of minority stress, namely external events and conditions that cause stress; the expectation of such events and conditions and the vigilance that this can create in the minority individual; and the internalization of stigma directed toward sexual minorities [54]. Minority stress theory actually provides limited insight into how and why these stressors undermine mental health outcomes in minority groups. This has conversely been a key focus of identity process theory. Jaspal and colleagues [44,52] have shown that minority stressors that cause identity threat, that is, those that succeed in curtailing feelings of self-esteem, self-efficacy, positive distinctiveness and self-efficacy, tend to be associated with greater levels of distress, anxiety and depression. It seems important, therefore, to measure not only reported exposure to stressors and which ones but also the ways in which they impact on identity processes in gay and bisexual men.

Minority stress theory notes that people attempt to cope with minority stress in varied ways. However, the theory does not allow us to develop predictions about specific coping responses that may be deployed by gay and bisexual men who face minority stress.

## 7. Coping

Identity process theory posits that the experience of identity threat will activate coping responses [8]. People possess different coping styles, that is, “clusters of coping strategies that they tend to use in conjunction as a habitual response to particular types of identity threat” [55] (p. 225). Specific strategies that operate within these coping styles are varied and are manifested at multiple levels. They may be intrapsychic, residing at the level of the individual; interpersonal, focusing on changing relationships with other people; or intergroup, relying on changes to relationships with particular social groups. Sometimes various strategies operating at distinct levels are activated in response to a threat to identity. The multitude of coping styles, strategies and tactics cannot all be summarized in this article but some of the key strategies found in previous research into gay men’s health and well-being are highlighted as illustrative examples.

### 7.1. Intrapsychic Strategies

At an intrapsychic level, people facing identity threat may simply deny that a threat exists. This amounts to deflection of the threat. For instance, some gay and bisexual men who are diagnosed with HIV, which can jeopardize multiple principles of identity simultaneously, may initially react by denying this reality or its significance. They may simply not acknowledge or internalize the news being communicated to them. This reflects a strategy for protecting self-esteem, continuity, positive distinctiveness and self-efficacy [56]. After all, an event, experience or situation can threaten identity only if it is allowed to gain access to consciousness. Events, situations and practices that facilitate denial are likely to be sustained so that the individual can continue to deny. Chemsex has frequently been described as a form of psychological escapism, possibly because it enables participants transiently to evade stressors that cause identity threat [57]. This can include forgetting about one’s positive HIV status. In his qualitative study of chemsex participants, Jaspal [58] describes the psychological manifestations of escapism in chemsex, namely transient depersonalization (in some cases, out-of-body experiences) and fantasizing about ideal versions of the self. It is easy to see how both of these psychological strategies can support the overarching coping response of denial. Although denial can have short-term benefits for the individual, enabling them temporarily to deflect threats to identity, this coping strategy is generally not sustainable as its efficacy tends to wane over time and the individual may eventually be required to confront the original hazard to identity. 

People may also engage in acceptance strategies, such as anticipatory re-structuring. This can be thought of as a pre-emptive strategy for managing identity threat by introducing modifications to the identity structure in anticipation of the threat. For instance, a gay or bisexual man who is aware of their engagement in sexual risk behaviors may anticipate that they have been infected with HIV and, prior to receiving their HIV test result, may initiate changes in the identity structure to accommodate a possible HIV diagnosis. They may begin to inform themselves about HIV, think about the implications of a positive serostatus for their relationships with others and connect with other people living (and thriving) with HIV. All these tactics could, of course, expose the individual to more favorable social representations of HIV and enable them to assimilate-accommodate, and evaluate more positively, their HIV status, transforming it from a hazard into something more manageable for identity, see [37]. Gay and bisexual men who have successfully assimilated-accommodated their positive HIV status and who evaluate it more positively may not perceive the need to engage in escapism and may therefore be less inclined to engage in chemsex, although this will of course depend on their own reasons for doing so.

### 7.2. Interpersonal Strategies

At an interpersonal level, individuals may isolate themselves from others in order to avert or alleviate threats to identity. Like denial, this constitutes a type of deflection strategy since it enables the individual to reduce contact with others who may (re-)ignite the threat to identity. For instance, internalized homonegativity is associated with greater identity threat [50] and may spur gay and bisexual men to isolate themselves both from heterosexual people and from other gay and bisexual men [59]. Gay and bisexual men often refer to the anonymity of their sexual encounters when practicing chemsex—they do not necessarily know, or get to know, others on the chemsex scene and are able to derive sexual satisfaction with limited social interaction. Of course, isolation constitutes a short-term strategy that can avert immediate threats to identity but it is not sustainable or adaptive, primarily because it reduces the individual’s ability to derive social support from others. The derivation of social support, conversely, is known to be adaptive and effective in the long term [9,60].

Self-disclosure is the antithesis of isolation. This strategy enables the individual to exchange confidences with a trusted other about the source of their identity threat, which can provide them with the opportunity to obtain positive feedback, validate aspects of their identity and make sense of the source of the threat. In other words, it is a facilitator of social support. People who self-disclose may come to view the hazard differently, thereby enabling them to re-construe it as something more manageable. For instance, in their study of Colombian gay men living with HIV [61], it was found that some participants attributed their homosexuality to adverse childhood experiences, such as sexual abuse. This attribution tendency essentially reflected their internalized homonegativity given that they perceived their homosexuality as a maladaptive response to these adverse experiences. They believed that, had they not had these adverse experiences, they would be heterosexual. However, self-disclosure could enable them to re-construe the meaning of their sexual identity, to evaluate it in more positive terms and thus to assimilate and accommodate it within the identity structure. Given that internalized homonegativity appears to be associated with chemsex participation [62], self-disclosure may reduce the likelihood of chemsex engagement as a strategy for alleviating the psychological effects of internalized homonegativity.

### 7.3. Intergroup Strategies

At an intergroup level, people may make use of their multiple group memberships strategically. They may attenuate the significance of some group memberships and accentuate that of others. For instance, gay and bisexual men who anticipate identity threat on the basis of their sexuality may attenuate the salience of their sexuality (as a group membership) in some contexts and instead foreground another group membership. This has been shown in relation to the management of sexuality and religion among gay men of religious faith, especially where there is scope for perceived incompatibility or in contexts in which stigma from others is anticipated [51]. In some respects, this amounts to a form of deflection as it implies the individual’s refusal to acknowledge a group membership that is stigmatized. They transiently forget that the stigmatized group membership exists which can provide temporary protection from identity threat. It is noteworthy that this strategy is not invariably maladaptive as individuals may strategically shift between group memberships, simply emphasizing some over others in particular contexts while retaining an awareness of them all, see [51]. Like denial and isolation, multiple group memberships may require a psychological escape (e.g., chemsex) in order to be sustained.

A clearly adaptive strategy is that of engaging in group action, itself a corollary of self-disclosure and particularly the derivation of social support. By engaging in group action, gay and bisexual men seek to challenge the social, cultural and/or political status quo that causes identity threat and to promote social change to facilitate lasting benefits for their own identities and those of others who share their predicament. The strategy can be defined as intergroup because it involves an “us versus them” mentality whereby the ingroup is mobilized to challenge an outgroup perceived to be hostile toward their interests. For instance, LGBT activism has clearly produced benefits for gay and bisexual men (and indeed other subgroups within the LGBT category) because it provides a sense of solidarity, on the one hand, and enables members of this diverse group to promote positive changes to legislation, social attitudes and, thus, to their own identities. The perception of these positive changes can serve to challenge (internalized) homonegativity (itself associated with identity threat) but also to reduce the risk of identity threat in others in their ingroup and in future generations who will, as a consequence, enjoy more favorable social and psychological conditions for their identity construction and enactment. This strategy might benefit self-esteem, positive distinctiveness, self-efficacy and continuity in those who adopt it. Moreover, activists tend to describe a strong sense of purpose as well as a connection with others [63]. Those who engage in group action may not perceive the need to escape and disconnect from their reality because they have embraced it and are actively doing something to promote positive change for themselves and others.

### 7.4. The Significance of Identity Resilience in Coping

People generally have particular coping styles that they use in response to particular types of threat [55]. The Coping with Identity Threat Scale has been developed to measure these coping styles. There is a burgeoning literature on the factors that predict coping styles, which generally point to the significance of personality traits, previous experiences and social context, for example, [64,65]. 

Identity resilience appears to constitute an important predictor of coping response. Identity resilience refers to the individual’s subjective overall appraisal of their self-esteem, self-efficacy, positive distinctiveness and continuity. Of course, an individual’s levels of these principles will fluctuate over time and in response to particular events, situations and experiences but they generally possess an overall perception of their identity resilience when reflecting on their life experiences. A person’s past and current circumstances will determine their identity resilience. In recent research using identity process theory [66,67,68], it has been found that identity resilience is associated with the deployment of more adaptive and sustainable coping strategies. A person with relatively higher levels of identity resilience may feel empowered to deploy coping strategies that are potentially more challenging, such as anticipatory re-structuring, self-disclosure, the exchanging of confidences, the derivation of social support and engagement in group action. This can be attributed to the higher resilience of their identity in the face of possible risks associated with these more adaptive strategies, such as rejection from others, denigration and stigma. Incidentally, it has also been found that higher baseline levels of identity resilience are actually associated with lower levels of identity threat upon exposure to a hazard, such as stressors associated with one’s sexuality [44,50]. More generally, the application of an effective resilience framework has been deemed to be useful for promoting positive behavior change in the context of chemsex [69].

## 8. Putting the Pieces Together: Stressors, Identity Threat and Coping

In recent years, many empirical studies of chemsex participation have been conducted in many countries, for example, [7,58,70]. Epidemiological and sexual health research tends to examine the correlates of engagement in chemsex, with a view to understanding the (sexual) health risks associated with the practice as well as to develop a “profile” of the average chemsex participant [71]. This research is valuable as it enables practitioners to appraise the relevant risks in an evidence-based manner and potentially to intervene proactively before the hazard materializes. 

Like the present article, many of these studies seek to understand why gay and bisexual men actually engage in chemsex. In the psychological literature [9,58], there has been a key focus on the specific stressors that they face and their relationship with chemsex participation. In her reflections on therapeutic encounters with chemsex participants, Evans [72] notes the social pressures of conformity to particular body types, physical appearance and masculinity norms, which can undermine feelings of self-esteem among those who fail to conform. She notes that this decreased self-esteem can lead to the negative emotional experience of shame. People may be facing identity threat and chemsex may provide an effective escape from this negative psychological experience. Furthermore, the toxicological effects of chemsex are also relevant to identity processes. Mephedrone and methamphetamine tend to induce feelings of euphoria and sexual self-confidence [73], which may transiently enhance self-esteem and self-efficacy, respectively. It may also impair memory performance [74], which could actually reduce the individual’s consciousness of events, experiences and situations (e.g., stressors) that previously challenged their sense of continuity. The toxicological effects of chemsex for identity processes will require further investigation.

Chronic prejudice due to one’s sexual orientation (and its corollary, internalized homonegativity) can lead to feelings of distress, which reflects poor mental health [52]. Chronic prejudice not only increases the risk of internalized homonegativity, whereby gay and bisexual men may find it difficult to develop, sustain and nurture intimate relationships, but also stimulates fear of rejection. They may come to anticipate and perceive stigma and rejection even in innocuous situations. Similarly, Pollard et al. [62] note that stressors associated with homonegativity and with navigating the “gay scene” can lead to feelings of marginalization and loneliness, thereby rendering chemsex an attractive means of escaping these stressors. For many, chemsex provides a psychologically safe space in which internalized homonegativity can be compartmentalized, that is, put to one side in the identity structure, and a sense of intimacy and connection can be established with other gay and bisexual men, however transiently. Indeed, as the work of Pollard et al. [62] (p. 420) shows, the perception that the chemsex environment enhances identity (or at least protects it from threat) can make self-withdrawal from this social environment and the return to “a marginalized mainstream life with limited opportunity for connection with gay men” difficult for some gay and bisexual men. 

Drawing upon the notion of syndemics, Pollard et al. [62] argue that chemsex may constitute a maladaptive strategy for coping with the negative emotions that arise from some of the prime stressors discussed in this article—most notably, homonegativity and the shame that often accompanies exposure to this stressor. After all, internalized homonegativity is positively correlated with identity threat. Conversely, the chemsex environment can operate as a liberating social and psychological space, which enables participants to escape compulsory heterosexuality and heteronormativity that pervade mainstream society [75] and thus to cope. Qualitative research into gay and bisexual men’s “narratives of pleasure” in relation to chemsex emphasizes the social, psychological and emotional functions performed by the practice and may therefore be useful in enabling us to understand how chemsex operates as a coping strategy [23]. In short, when chemsex operates as an effective coping strategy (however short-term the benefits may be) and particularly when it is actually perceived to enhance identity processes, it is likely to be utilized by the individual to protect identity.

## 9. The Implications of Chemsex for Sexual Health

Notwithstanding the legitimate concerns expressed by some scholars and commentators about the potential pathologization of chemsex, for example, [15], there is consensus in sexual health research that the negative implications of chemsex for sexual health outcomes among gay and bisexual men are significant. 

A systematic review of research articles from high-income countries, which appeared between January 2000 and September 2018 focusing on the use of chemsex drugs before or during sex, found that chemsex participants were more likely to report engagement in condomless anal intercourse than those who reported no engagement in chemsex [7]. In their analysis of survey data from 2014, Pufall et al. [76] found that, in the previous year, 29.5% of 392 sexually active participants reported having engaged in chemsex and that 10.1% had injected drugs. Engagement in chemsex was associated with increased odds of engaging in condomless anal intercourse and with engaging in serodiscordant anal intercourse with a detectable HIV viral load and with increased odds of having had an STI diagnosis. 

Similarly, in a case notes review study of all gay, bisexual and other men who have sex with men attending two South London clinics from 1 June 2014 and 31 January 2015, Hegazi et al. [20] found that those who were living with HIV were more likely to report chemsex and that men who reported chemsex had a higher incidence of acute bacterial STIs, rectal STIs or hepatitis C. In terms of behavioral correlates, they found that chemsex was associated with having a higher number of sexual partners and increased odds of engaging in various risk behaviors, including transactional sex, group sex, fisting, sharing sex toys and injecting drug use. Crucially, chemsex participants were more likely to have accessed post-exposure prophylaxis for HIV in the study period. In their cross-sectional online survey of 2328 gay and bisexual men in Scotland, Wales, Northern Ireland and the Republic of Ireland, Frankis et al. [27] found that, while prior experience of engaging in chemsex was not prevalent (just 18% of the entire sample and only 6.1% who reported doing chemsex in the last 12 months), this was positively associated with engagement in a variety of high-risk sexual behaviors, including being fisted, having had sex in exchange for goods and being HIV-positive. 

These are illustrative studies demonstrating the sexual health risks of chemsex. On the whole, chemsex participants are more likely to engage in behaviors that increase their risk of acquiring and transmitting HIV and other STIs. Moreover, many of the gay and bisexual men who engage in chemsex report dissatisfaction with sober sex, with some noting their inability to engage in sex without substances [77]. Some also report dissatisfaction with the mainstream gay culture that awaits them outside of chemsex environments where the stressors of heteronormativity, rejection, judgment and prejudice may reignite the threats to identity that are clearly attenuated for some in the context of chemsex. Thus, some gay and bisexual men may experience challenges in constructing a positive sexual identity in the absence of substances. 

It is noteworthy that, even during the pandemic, chemsex continued to take place [78]. However, some gay and bisexual men did seek to reduce their risk of poor sexual health. For instance, Møller [79] has described the unusual practice of digital chemsex, that is, the congregation of gay men in virtual settings where they consume substances and engage in masturbatory practices and verbal sexual activity in a voyeuristic and exhibitionist manner. This indicates that some gay and bisexual men were thinking about risk reduction while engaging in chemsex. The key is to understand why some gay and bisexual men strive to protect their (sexual) health while others do not. The available evidence would suggest that identity resilience acts as a buffer. This hypothesis is consistent with the notion that higher self-esteem is associated with greater self-care [80] and that higher self-efficacy enables individuals to take proactive steps to protect themselves from health risks [81].

## 10. Conclusions

Chemsex appears to be fairly widespread in gay and bisexual communities in major cities around the world. It is facilitated by the use of geospatial social networking applications in this population. The practice is sustained because of the powerful psychological functions that it performs for many of those who engage in it—most notably for identity processes [58,82]. Although some gay and bisexual men do manage their chemsex engagement effectively, it is evident that the practice presents major risks to psychological and sexual health among many others [83,84]. Indeed, focusing HIV prevention efforts on gay and bisexual men reporting chemsex behavior would be especially beneficial from a public health perspective [85]. Complementing contemporary models of health behavior from public health research, the approach outlined in this article focuses on the psychological motivations underpinning the individual’s behavior. It is intended to be used in conjunction with more socially oriented accounts of chemsex. It will be valuable to integrate emerging social constructionist perspectives that focus on how chemsex is represented, talked about and re-construed in different social contexts [15,16,79,86].

A key aim of this article was to examine the potential motivations of both gay and bisexual men who engage in chemsex and its possible health consequences. There is much evidence that chemsex is associated with engagement in sexual risk behaviors and, consequently, with increased risk of HIV infection and transmission. Despite the risks involved, some gay and bisexual men continue to engage in the practice. Identity process theory provides an integrative framework that can enable researchers to predict the social and psychological circumstances under which individuals will engage in chemsex. As highlighted in this article, much research shows that gay and bisexual men face a multitude of distal and proximal stressors across the life course (e.g., homonegativity, internalized homonegativity, body image concerns), which can heighten the risk of identity threat. Identity threat itself is harmful to psychological well-being and therefore prompts coping in the threatened individual. Some of these strategies are adaptive but many are not. The determinants of these strategies depend on a number of factors—psychological, interpersonal, social and institutional [69].

The available evidence would suggest that, in the face of identity threat, gay and bisexual men may engage in chemsex as a coping response that can facilitate and enhance various maladaptive strategies and tactics, relying mainly on deflection. This is consistent with the long-standing empirical observation that chemsex operates as a form of psychological escapism for many of the gay and bisexual men who practice it [57]. The toxicological effects of chemsex play a significant role. Given the lure of these strategies (especially as they provide transient boosts to the identity principles), individuals may be motivated to engage in chemsex and to sustain it over time. In view of the observation that some gay and bisexual men who practice chemsex no longer feel able to enjoy sober sex, it is hypothesized that the prospect of renouncing the practice may itself become threatening for identity. 

A second aim of the article was to identify the protective factors that may limit the risk of engagement in problematic chemsex. On the basis of the research reviewed, it seems important to encourage gay and bisexual men to adopt more sustainable, adaptive coping strategies that can avert or alleviate threat, thereby obviating the perceived need to resort to chemsex as a coping response. Those gay and bisexual men who have decreased access to sustainable adaptive coping strategies, namely those contingent on acceptance of the threat, may be more susceptible to engaging in chemsex. Societal and psychotherapeutic interventions for enhancing psychological well-being among gay and bisexual men should focus on building feelings of identity resilience. It is hypothesized that identity resilience will reduce the perceived need to engage in chemsex for some and promote safer approaches to chemsex in others. The promotion of identity resilience in clients will require a person-centered therapeutic approach that can enable the individual to focus on positive aspects of their lives that generate feelings of self-esteem, self-efficacy, continuity and positive distinctiveness [9]. Having a relatively high level of identity resilience is likely to provide access to more sustainable adaptive coping strategies among those facing, or at risk of, identity threat. Individual interventions of this kind could also be delivered effectively in digital settings [87].

Although there are evident risks to psychological and sexual health, chemsex may be perceived as enhancing the very identity principles that are susceptible to threat among gay and bisexual men exposed to sexuality-related stressors. This will provide a strong psychological incentive for the individual to (continue to) engage in chemsex and to resist self-withdrawal from the practice. A key first step to self-withdrawal from chemsex settings is of course help-seeking [72]. Some commentators are now calling for the de-stigmatization and de-pathologization of chemsex. The benefits of doing so are clear. People are more likely to seek support if they are confident that they will not be stigmatized or pathologized by others. The long-standing stigma surrounding drug use and homosexuality and the emerging stigmatization of chemsex in particular can inhibit the assimilation-accommodation of the practice in identity among gay and bisexual men, thereby reducing their willingness to acknowledge their chemsex participation and its risks to their health and to seek support in relation to the practice when this is needed. In this regard, the social construction of chemsex is of particular significance—how it is represented and talked about will shape how it is understood [16].

This article outlines a program of empirical psychological research needed to facilitate a better understanding of why gay and bisexual men engage in chemsex, why some sustain this practice and how they can be better supported. The hypotheses outlined here should be tested using a variety of methods. Some can be addressed using a cross-sectional survey design while others will require experimental and longitudinal approaches. Qualitative approaches that can shed light on the social construction of chemsex—in health communication literature as well as in individuals’ accounts of the phenomenon—will be valuable. Mixed methods research has an important role to play in addressing the challenging questions that this article raises. Interdisciplinary research will be vital—psychologists must collaborate with toxicologists, physicians and public health experts. As Fish et al. [88] note, evidence-based health and well-being interventions that are underpinned by theory and that take into account the total identity of the individual and group concerned are more likely to be effective in the longer term.

## Data Availability

Not applicable.
